# Supplementary material for: CRISPR-Cas9 for selective targeting of somatic mutations in pancreatic cancers
Source: NAR Cancer. 2024 Jun 19;6(2):zcae028. doi: 10.1093/narcan/zcae028 (PMC11195629; doi:10.1093/narcan/zcae028)
Supplement: zcae028_Supplemental_Files [file zcae028_supplemental_files.zip › Figure S1-8.pptx]

## Slide 1
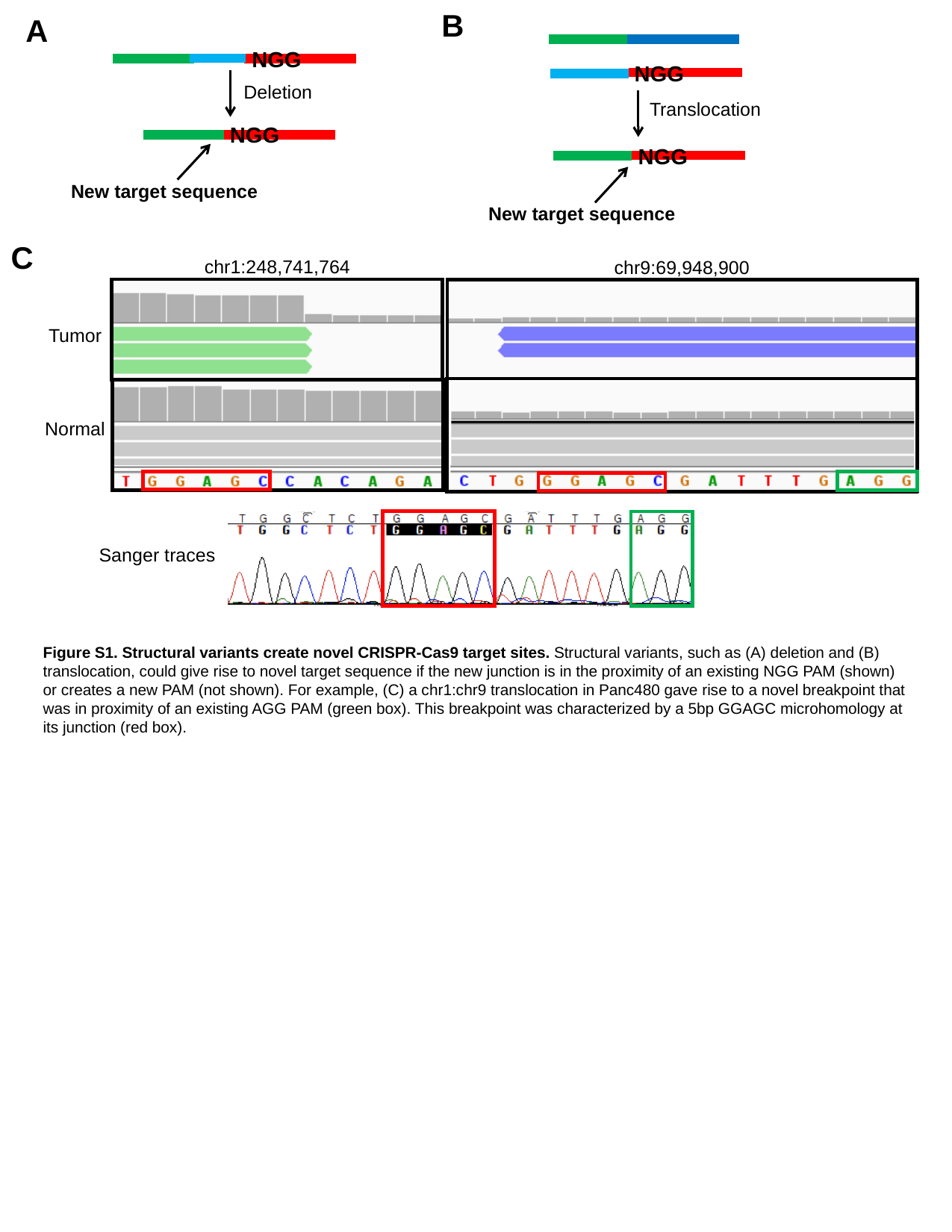

B
A
NGG
NGG
Deletion
Translocation
NGG
NGG
New target sequence
New target sequence
C
chr1:248,741,764
chr9:69,948,900
Tumor
Normal
Sanger traces
Figure S1. Structural variants create novel CRISPR-Cas9 target sites. Structural variants, such as (A) deletion and (B) translocation, could give rise to novel target sequence if the new junction is in the proximity of an existing NGG PAM (shown) or creates a new PAM (not shown). For example, (C) a chr1:chr9 translocation in Panc480 gave rise to a novel breakpoint that was in proximity of an existing AGG PAM (green box). This breakpoint was characterized by a 5bp GGAGC microhomology at its junction (red box).

## Slide 2
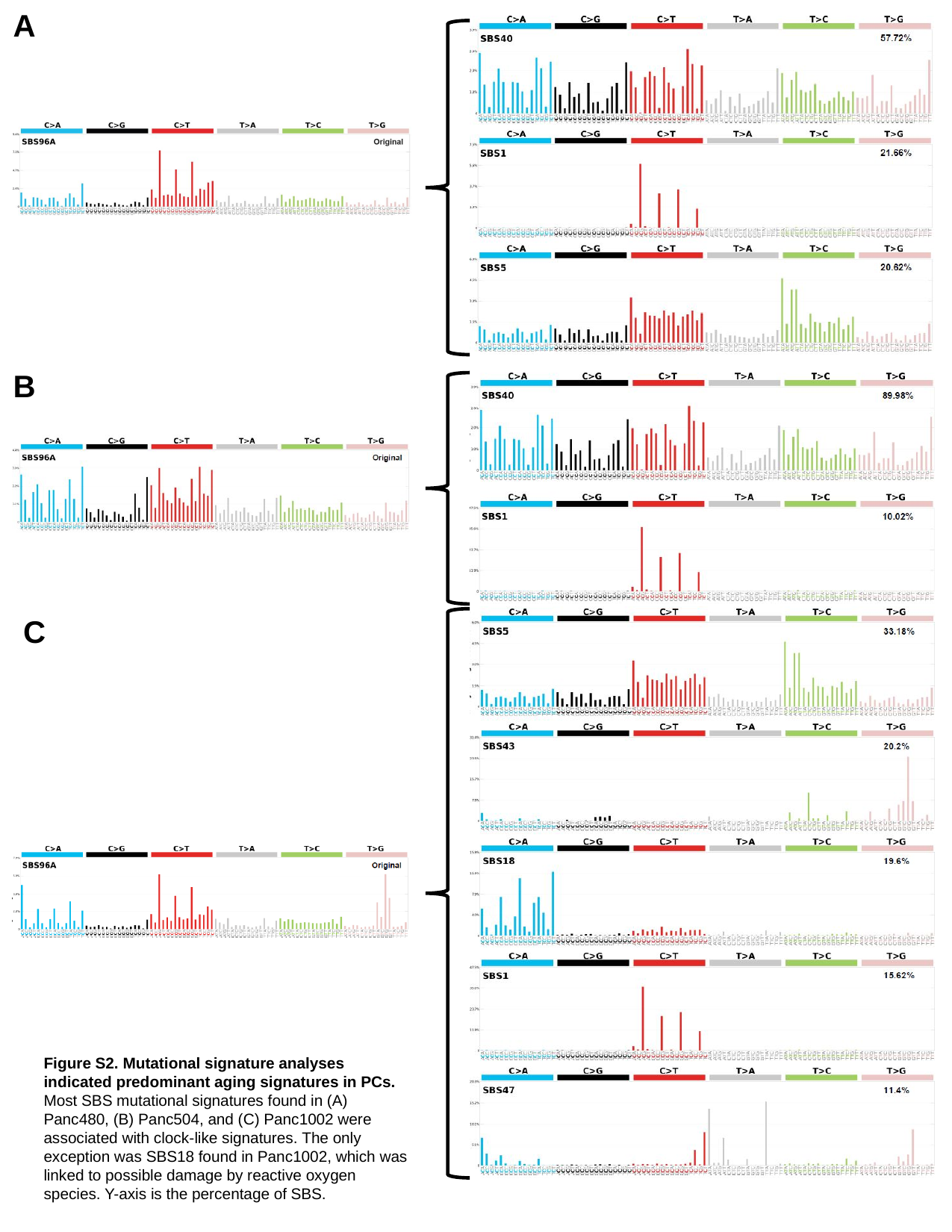

A
B
C
Figure S2. Mutational signature analyses indicated predominant aging signatures in PCs. Most SBS mutational signatures found in (A) Panc480, (B) Panc504, and (C) Panc1002 were associated with clock-like signatures. The only exception was SBS18 found in Panc1002, which was linked to possible damage by reactive oxygen species. Y-axis is the percentage of SBS.

## Slide 3
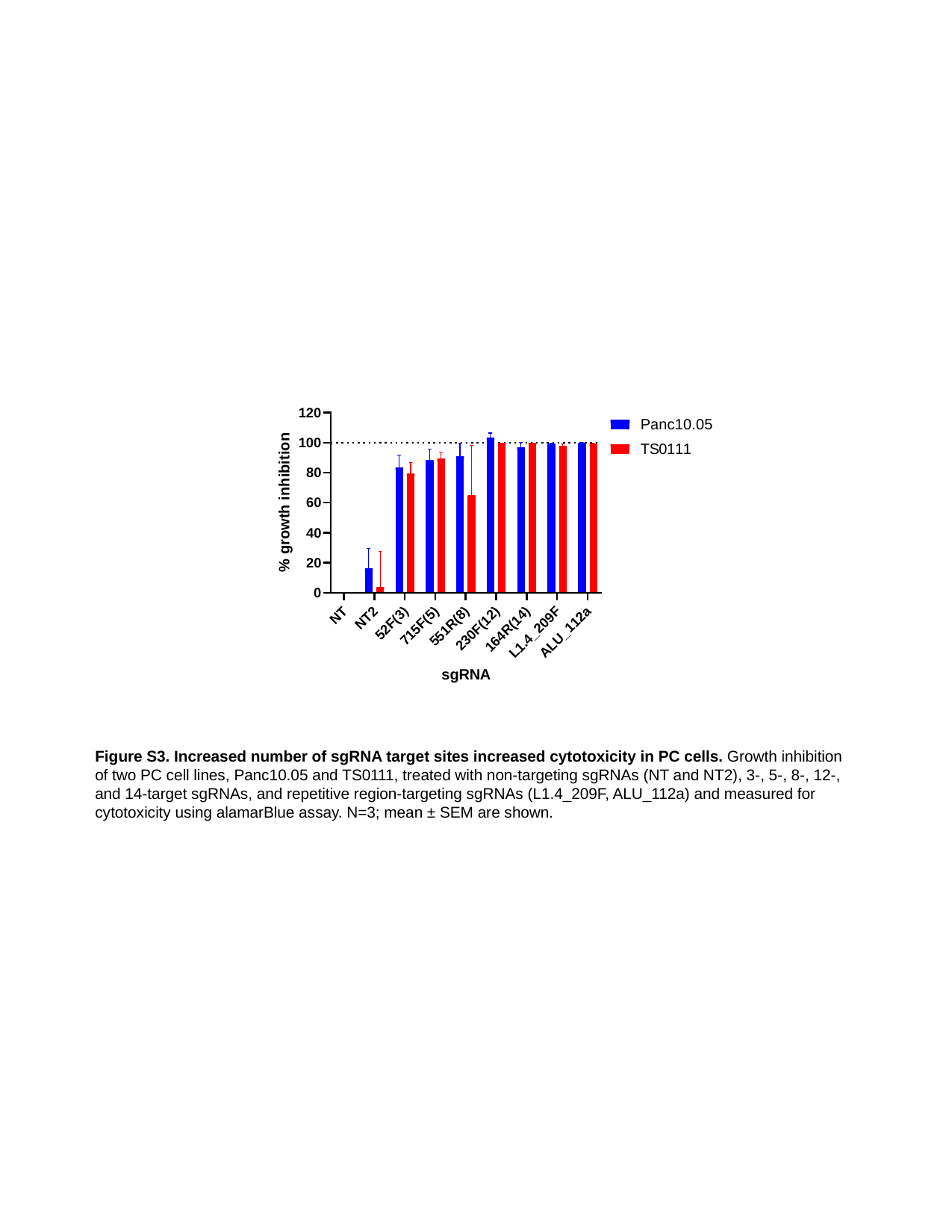

Figure S3. Increased number of sgRNA target sites increased cytotoxicity in PC cells. Growth inhibition of two PC cell lines, Panc10.05 and TS0111, treated with non-targeting sgRNAs (NT and NT2), 3-, 5-, 8-, 12-, and 14-target sgRNAs, and repetitive region-targeting sgRNAs (L1.4_209F, ALU_112a) and measured for cytotoxicity using alamarBlue assay. N=3; mean ± SEM are shown.

## Slide 4
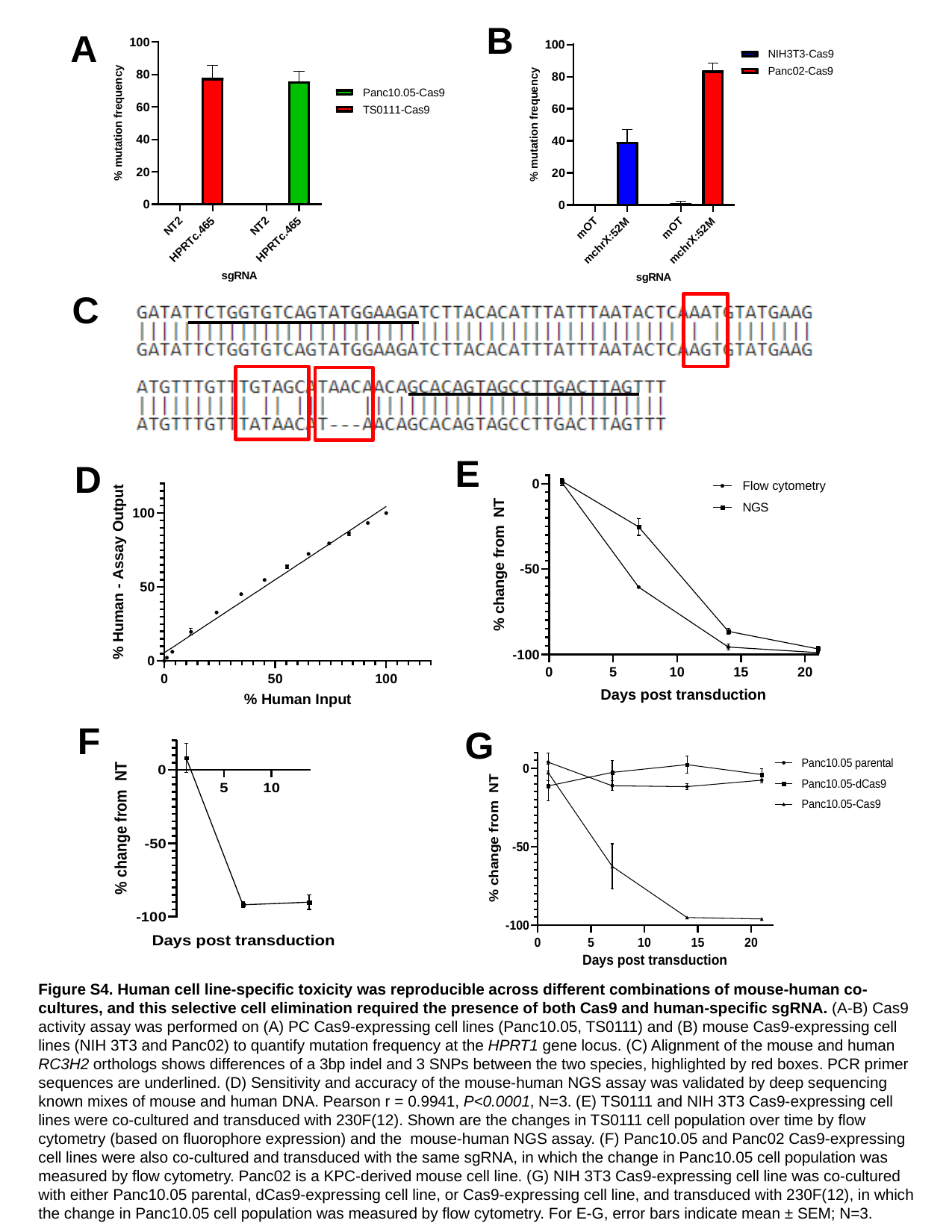

B
A
C
E
D
F
G
Figure S4. Human cell line-specific toxicity was reproducible across different combinations of mouse-human co-cultures, and this selective cell elimination required the presence of both Cas9 and human-specific sgRNA. (A-B) Cas9 activity assay was performed on (A) PC Cas9-expressing cell lines (Panc10.05, TS0111) and (B) mouse Cas9-expressing cell lines (NIH 3T3 and Panc02) to quantify mutation frequency at the HPRT1 gene locus. (C) Alignment of the mouse and human RC3H2 orthologs shows differences of a 3bp indel and 3 SNPs between the two species, highlighted by red boxes. PCR primer sequences are underlined. (D) Sensitivity and accuracy of the mouse-human NGS assay was validated by deep sequencing known mixes of mouse and human DNA. Pearson r = 0.9941, P<0.0001, N=3. (E) TS0111 and NIH 3T3 Cas9-expressing cell lines were co-cultured and transduced with 230F(12). Shown are the changes in TS0111 cell population over time by flow cytometry (based on fluorophore expression) and the mouse-human NGS assay. (F) Panc10.05 and Panc02 Cas9-expressing cell lines were also co-cultured and transduced with the same sgRNA, in which the change in Panc10.05 cell population was measured by flow cytometry. Panc02 is a KPC-derived mouse cell line. (G) NIH 3T3 Cas9-expressing cell line was co-cultured with either Panc10.05 parental, dCas9-expressing cell line, or Cas9-expressing cell line, and transduced with 230F(12), in which the change in Panc10.05 cell population was measured by flow cytometry. For E-G, error bars indicate mean ± SEM; N=3.

## Slide 5
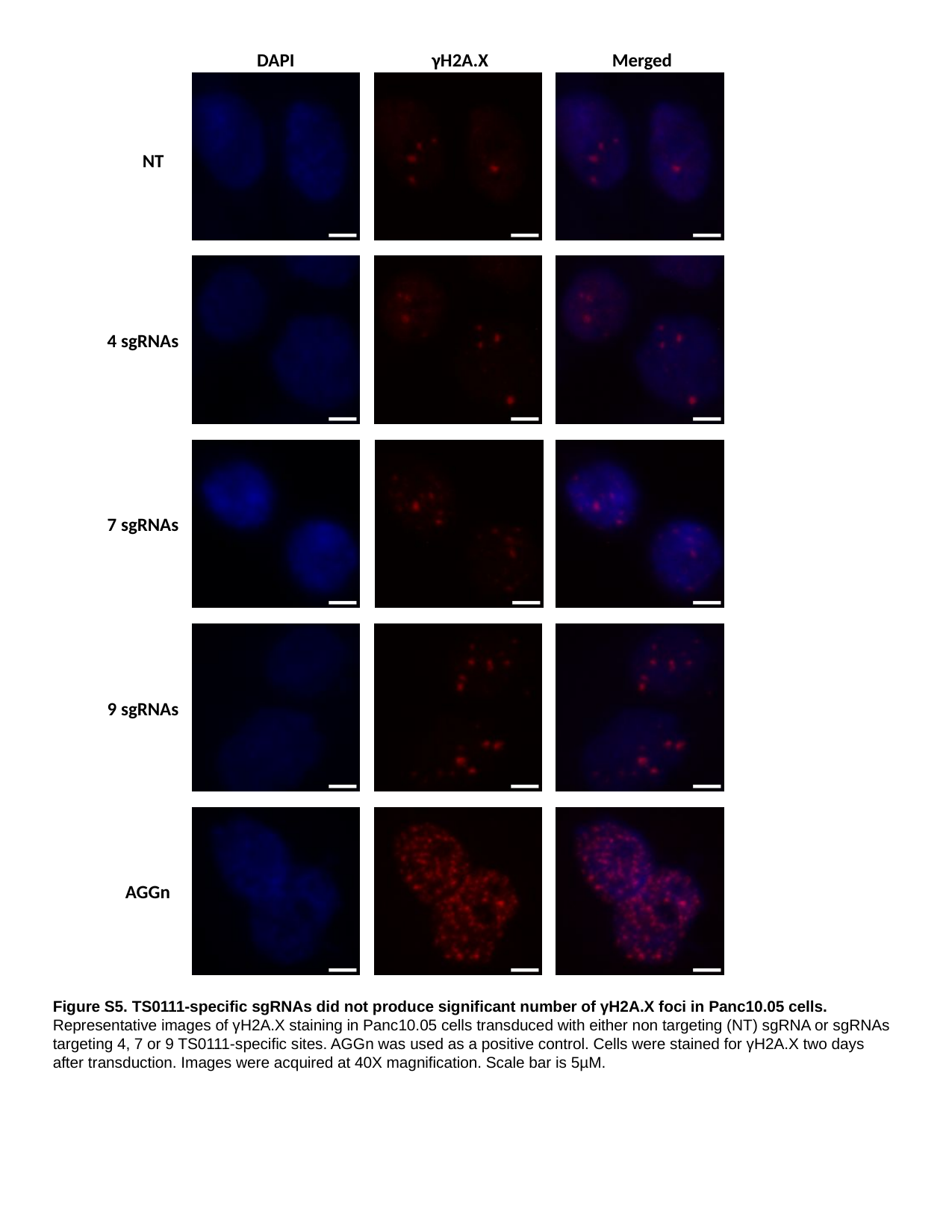

Merged
DAPI
γH2A.X
NT
4 sgRNAs
7 sgRNAs
9 sgRNAs
AGGn
Figure S5. TS0111-specific sgRNAs did not produce significant number of γH2A.X foci in Panc10.05 cells. Representative images of γH2A.X staining in Panc10.05 cells transduced with either non targeting (NT) sgRNA or sgRNAs targeting 4, 7 or 9 TS0111-specific sites. AGGn was used as a positive control. Cells were stained for γH2A.X two days after transduction. Images were acquired at 40X magnification. Scale bar is 5µM.

## Slide 6
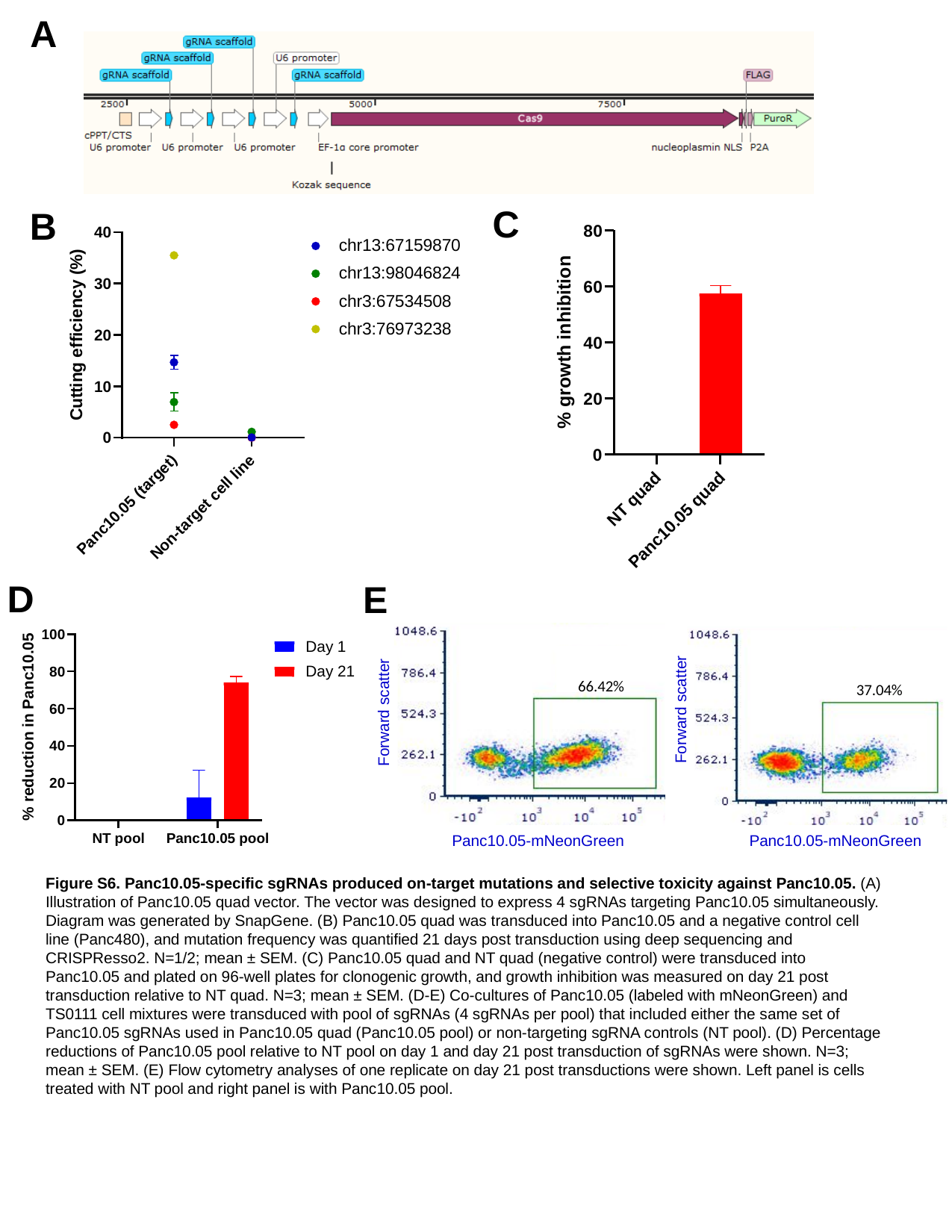

A
C
B
D
E
66.42%
37.04%
Forward scatter
Forward scatter
Panc10.05-mNeonGreen
Panc10.05-mNeonGreen
Figure S6. Panc10.05-specific sgRNAs produced on-target mutations and selective toxicity against Panc10.05. (A) Illustration of Panc10.05 quad vector. The vector was designed to express 4 sgRNAs targeting Panc10.05 simultaneously. Diagram was generated by SnapGene. (B) Panc10.05 quad was transduced into Panc10.05 and a negative control cell line (Panc480), and mutation frequency was quantified 21 days post transduction using deep sequencing and CRISPResso2. N=1/2; mean ± SEM. (C) Panc10.05 quad and NT quad (negative control) were transduced into Panc10.05 and plated on 96-well plates for clonogenic growth, and growth inhibition was measured on day 21 post transduction relative to NT quad. N=3; mean ± SEM. (D-E) Co-cultures of Panc10.05 (labeled with mNeonGreen) and TS0111 cell mixtures were transduced with pool of sgRNAs (4 sgRNAs per pool) that included either the same set of Panc10.05 sgRNAs used in Panc10.05 quad (Panc10.05 pool) or non-targeting sgRNA controls (NT pool). (D) Percentage reductions of Panc10.05 pool relative to NT pool on day 1 and day 21 post transduction of sgRNAs were shown. N=3; mean ± SEM. (E) Flow cytometry analyses of one replicate on day 21 post transductions were shown. Left panel is cells treated with NT pool and right panel is with Panc10.05 pool.

## Slide 7
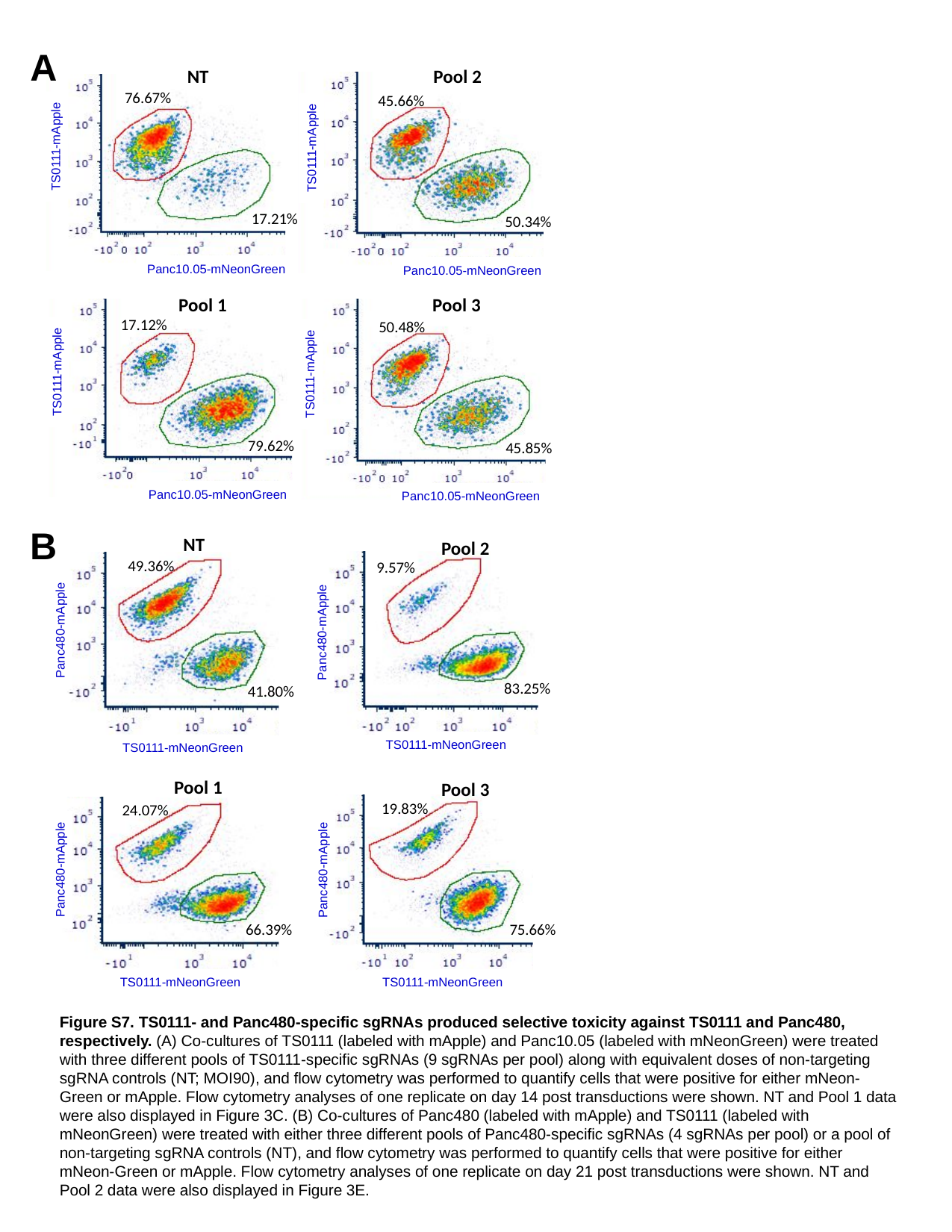

A
Pool 2
NT
76.67%
45.66%
TS0111-mApple
TS0111-mApple
17.21%
50.34%
Panc10.05-mNeonGreen
Panc10.05-mNeonGreen
Pool 1
Pool 3
17.12%
50.48%
TS0111-mApple
TS0111-mApple
79.62%
45.85%
Panc10.05-mNeonGreen
Panc10.05-mNeonGreen
B
NT
Pool 2
49.36%
9.57%
Panc480-mApple
Panc480-mApple
83.25%
41.80%
TS0111-mNeonGreen
TS0111-mNeonGreen
Pool 1
Pool 3
19.83%
24.07%
Panc480-mApple
Panc480-mApple
75.66%
66.39%
TS0111-mNeonGreen
TS0111-mNeonGreen
Figure S7. TS0111- and Panc480-specific sgRNAs produced selective toxicity against TS0111 and Panc480, respectively. (A) Co-cultures of TS0111 (labeled with mApple) and Panc10.05 (labeled with mNeonGreen) were treated with three different pools of TS0111-specific sgRNAs (9 sgRNAs per pool) along with equivalent doses of non-targeting sgRNA controls (NT; MOI90), and flow cytometry was performed to quantify cells that were positive for either mNeon-Green or mApple. Flow cytometry analyses of one replicate on day 14 post transductions were shown. NT and Pool 1 data were also displayed in Figure 3C. (B) Co-cultures of Panc480 (labeled with mApple) and TS0111 (labeled with mNeonGreen) were treated with either three different pools of Panc480-specific sgRNAs (4 sgRNAs per pool) or a pool of non-targeting sgRNA controls (NT), and flow cytometry was performed to quantify cells that were positive for either mNeon-Green or mApple. Flow cytometry analyses of one replicate on day 21 post transductions were shown. NT and Pool 2 data were also displayed in Figure 3E.

## Slide 8
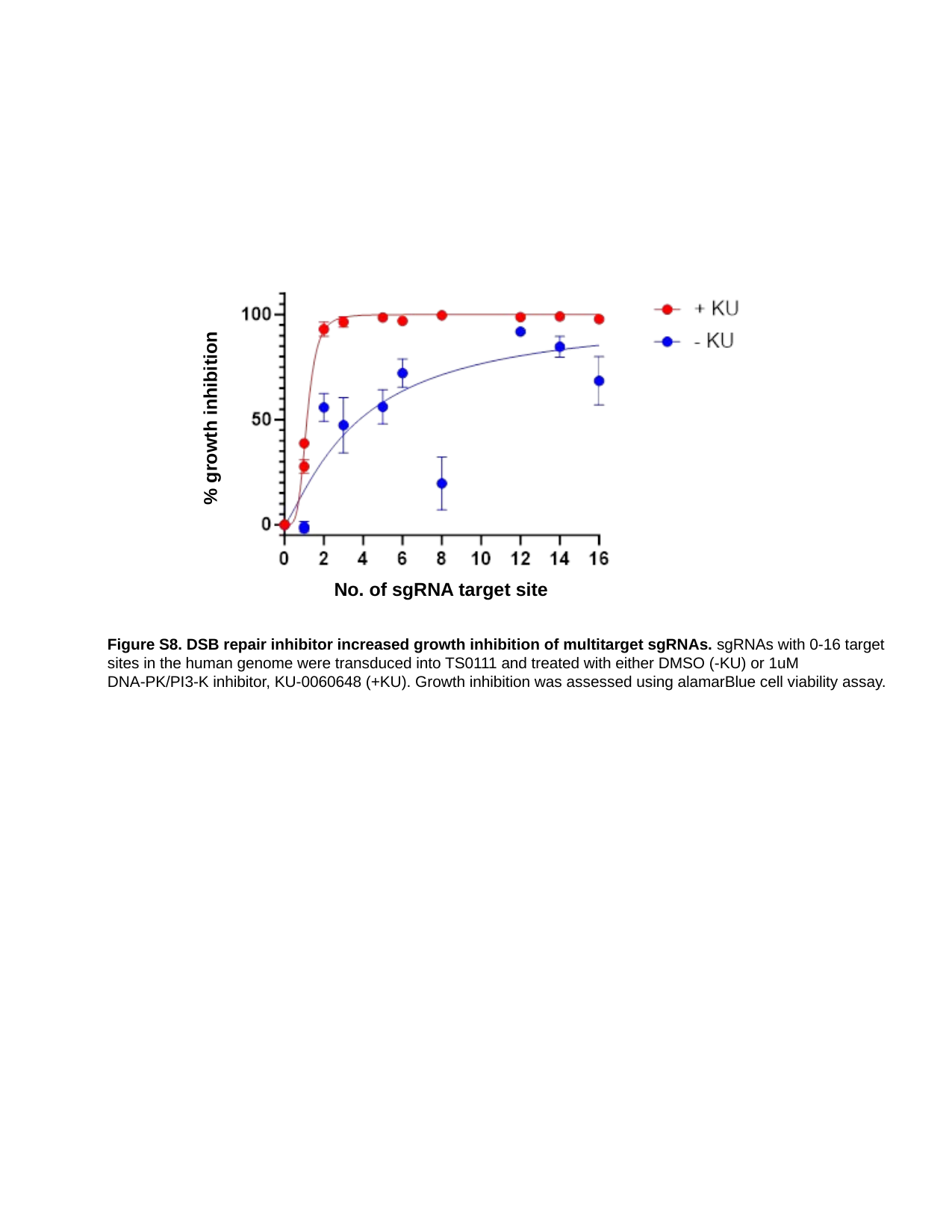

% growth inhibition
No. of sgRNA target site
Figure S8. DSB repair inhibitor increased growth inhibition of multitarget sgRNAs. sgRNAs with 0-16 target sites in the human genome were transduced into TS0111 and treated with either DMSO (-KU) or 1uM DNA-PK/PI3-K inhibitor, KU-0060648 (+KU). Growth inhibition was assessed using alamarBlue cell viability assay.
