# Supplementary material for: CRISPR-Cas9 for selective targeting of somatic mutations in pancreatic cancers
Source: NAR Cancer. 2024 Jun 19;6(2):zcae028. doi: 10.1093/narcan/zcae028 (PMC11195629; doi:10.1093/narcan/zcae028)
Supplement: zcae028_Supplemental_Files [file zcae028_supplemental_files.zip › Supplementary Table S1-11.docx]

**Supplementary Tables**

Table S1. Source of genomic DNA and mutation profile of the driver genes of three pancreatic cancer cases.

Table S2. Novel SVs discovered for sgRNA design.

Table S3. Novel PAMs discovered from SBSs using WGS.

Table S4. Novel PAMs discovered from SBSs using WES.

Table S5. Summary of base substitutions and somatic PAMs obtained from different ICGC projects.

Table S6. Multitarget sgRNAs.

Table S7. Number of target sites of sgRNAs for Cas9 activity and mouse-human co-culture assays in both mouse (mm10) and human (hg38) genomes.

Table S8. TS0111-specific sgRNAs for immunofluorescence staining and co-culture assays.

Table S9. sgRNAs used in co-cultures to test selectivity toxicity of Panc10.05-specific sgRNAs.

Table S10. sgRNAs used in co-cultures to test selectivity toxicity of Panc480-specific sgRNAs.

Table S11. Cutting efficiency and number of potential off-target sites of sgRNAs included in Panc480-MT7.

**Table S1. Source of genomic DNA and mutation profile of the driver genes of three pancreatic cancer cases.**

| **Sample** | **Source of tumor DNA** | **Source of normal DNA** | **Tumor *KRAS*** | **Tumor *CDKN2A*** | **Tumor *SMAD4*** | **Tumor *TP53*** |
| --- | --- | --- | --- | --- | --- | --- |
| Panc480 | Primary | Lymph | G12D | Frameshift | Homozygous deletion | V274A |
| Panc504 | Primary | Duodenum | G12V | Homozygous deletion | Homozygous deletion | Frameshift |
| Panc1002 | Primary | Lymph | Q61H | Homozygous deletion | Homozygous deletion | R248Q |

**Table S2. Novel SVs discovered for sgRNA design.**

| **Cell line** | **Total no. of somatic SVs** | **No. of Sanger-validated SVs** | **No. of SVs with PAM** | **No. of good sgRNAs^#^** |
| --- | --- | --- | --- | --- |
| Panc480 | 38 | 31 | 24 | 17 |
| Panc504 | 37 | 29 | 18 | 15 |
| Panc1002 | 31 | 30 | 25 | 18 |
| **Average** | **35** | **30** | **22** | **17** |

^#^ “Good sgRNA” is defined as sgRNAs that have >50 specificity score (prediction of how much the sgRNA sequence may lead to off-target cleavage) in CRISPOR. It includes sgRNAs that are inefficient (low knockout frequencies).

**Table S3. Novel PAMs discovered from SBSs using WGS.**

| **Cell line** | **No. of SBS** | **No. of somatic PAM^&^** | **% PAM** | **No. of PAM with VAF >95%** | **No. of good sgRNAs^#^** | **No. of Sanger-validated good sgRNAs** |
| --- | --- | --- | --- | --- | --- | --- |
| Panc480 | 4576 | 385 | 8.4 | 23 | 13 | 13 |
| Panc504 | 4502 | 417 | 9.3 | 76 | 48 | 47 |
| Panc1002 | 4566 | 448 | 9.8 | 78 | 38 | 37 |
| **Average** | **4548** | **417** | **9.2** | **63** | **33** | **32** |

^&^Somatic PAM indicates a SBS of NGN/NNG sequence to NGG (both + and - strands). Only mutations with a variant allele frequency (VAF) of at least 30% in tumor (to account for subclonal mutations that potentially arose from *in vitro* culture) and a minimum of 18X read depth in both normal and tumor were included.

^#^ “Good sgRNA” is defined as sgRNAs that have >50 specificity score (prediction of how much the sgRNA sequence may lead to off-target cleavage) in CRISPOR. It includes sgRNAs that are inefficient (low knockout frequencies).

**Table S4. Novel PAMs discovered from SBSs using WES.**

| **Cell line** | **Total no. of somatic mutations** | **No. of novel PAM** | **No. of good sgRNAs^#^** | **No. of good sgRNAs with PAM of VAF >95%** |
| --- | --- | --- | --- | --- |
| Panc480 | 44 | 8 | 5 | 2 |
| Panc504 | 38 | 3 | 0 | 0 |
| Panc1002 | 30 | 4 | 2 | 0 |
| **Average** | **37** | **5** | **2** | **1** |

^#^ “Good sgRNA” is defined as sgRNAs that have >50 specificity score (prediction of how much the sgRNA sequence may lead to off-target cleavage) in CRISPOR. It includes sgRNAs that are inefficient (low knockout frequencies).

**Table S5. Summary of tumor purity, base substitutions, and somatic PAMs obtained from different ICGC projects.**

| **Project** | **N** | **% tumor purity** | | **No. of base substitutions** | | **No. of somatic PAM** | | **% PAM^*^** | |
| --- | --- | --- | --- | --- | --- | --- | --- | --- | --- |
|  |  | **Median** | **IQR**^#^ | **Median** | **IQR**^#^ | **Median** | **IQR**^#^ | **Median** | **IQR**^#^ |
| APGI-AU | 44 | 29.7 | 29.2-40.1 | 5890.5 | 4058.8-8390.3 | 478.5 | 344.8-844.0 | 8.9 | 8.1-10.5 |
| PACA-CA | 130 | 38.2 | 29.8-47.8 | 5354.5 | 4232.8-7942.0 | 430.5 | 340.5-711.5 | 8.4 | 7.7-9.8 |
| LUCA-KR | 29 | 36.3 | 30.8-47.3 | 30553.0 | 19081.5-45893.0 | 2790.0 | 2211.5-3675.0 | 8.5 | 7.8-9.2 |
| OCCAMS-GB | 388 | 32.8 | 29.5-40.0 | 20106.0 | 13542.5-31705.0 | 3235.5 | 1741.3-6167.3 | 16.1 | 12.3-20.5 |
| **All** | **591** | **34.4** | **29.5-41.0** | **15552.0** | **7091.0-26989.0** | **2131.0** | **662.0-4535.0** | **12.9** | **9.0-18.2** |

^#^IQR indicates interquartile range (25^th^-75^th^ percentile).

*% PAM = No. of somatic PAM / No. of base substitutions

**Table S6. Multitarget sgRNAs.**

| **sgRNA** | **Sequence^1^** | **Number of perfect target sites (hg38)^2^** | **Number of potential off-target sites (hg38)^2^** | **Potential target sites in exons (hg38)^3^** | **Doench ’16 predicted efficiency score^4^** | **Function** |
| --- | --- | --- | --- | --- | --- | --- |
| NT | GTATTACTGATATTGGTGGG | 0 | 0-1-12 | 0-0-0-0 | NA | Negative control |
| NT2 | GCGAGGTATTCGGCTCCGCG | 0 | 0-0-2 | 0-0-0-0 | NA |  |
| 52F(3) | TAATTACTGCACGATGCGCA | 3 | 0-0-2 | 0-0-0-0 | 59 | Multitarget sgRNAs |
| 715F(5) | ATATATATGCGATCGAGCCC | 5 | 2-1-5 | 0-0-0-0 | 54 |  |
| 551R(8) | TTGAATTGAGTTGCAACCGA | 8 | 2-1-4 | 0-0-0-0 | 61 |  |
| 230F(12) | TTGTCCCACAATGATACTTG | 12 | 8-1-8 | 0-0-0-0 | 61 |  |
| 164R(14) | GGATATTTCACTACAGACTT | 14 | 5-2-15 | 0-0-0-0 | 53 |  |
| L1.4_209F | TGCCTCACCTGGGAAGCGCA | 604 | 939-1710-2213 | NA | 55 | Positive control |
| ALU_112a | TTGCCCAGGCTGGAGTGCAG | Repeat | NA**^5^** | NA | 58 |  |

1. Sequences are followed in the genome by canonical (NGG) and/or non-canonical (NGA/NAG) PAMs. 2. CRISPOR analysis of the sgRNAs to identify the potential perfect and off-target sites (1-2-3 mismatches) in the hg38 human reference genome. 3. Number of perfect and off-target sites (0-1-2-3 mismatches) that fall within exons. 4. Cutting efficiency score based on data trained by Doench *et al.* 2016. Recommended for sgRNAs expressed with U6 promoter. The higher the efficiency score, the more likely is cleavage at this position. 5. Not applicable.

**Table S7. Number of target sites of sgRNAs for Cas9 activity and mouse-human co-culture assays in both mouse (mm10) and human (hg38) genomes.**

| **sgRNA** | **Sequence^1^** | **No. of target site in hg38^2^**  **(0-1-2-3 mismatches)** | **No. of target site in mm10^2^**  **(0-1-2-3 mismatches)** | **Function** |
| --- | --- | --- | --- | --- |
| NT2 | GCGAGGTATTCGGCTCCGCG | 0-0-0-2 | NA^4^ | Cas9 activity assay |
| HPRTc.465 | TGGATTATACTGCCTGACCA | 1-0-2-8 | NA^4^ |  |
| mOT^3^ | GGGGCTGTACTGCTTAACCA | NA^4^ | 1-0-0-10 |  |
| mchrX:52M | TATACCTAATCATTATGCCG | NA^4^ | 1-0-0-7 |  |
| NT | GTATTACTGATATTGGTGGG | 0-0-1-12 | 0-0-3-6 | Mouse-human co-cultures |
| 230F(12) | TTGTCCCACAATGATACTTG | 12-8-1-8 | 0-0-1-13 |  |

1. Sequences are followed in the genome by canonical (NGG) and/or non-canonical (NGA/NAG) PAMs. 2. CRISPOR analysis of the sgRNAs to identify the potential perfect and off-target sites (1-2-3 mismatches) in the hg38 human reference genome. 3. Off-target sgRNA. 4. NA: not applicable.

**Table S8. TS0111-specific sgRNAs for immunofluorescence (IF) staining and co-culture assays.**

| **Target** | **sgRNA sequence** | **PAM** | **Location type** | **Potential off-target sites (0-1-2-3 mismatches) in hg38^*^** | **IF group** | **Co-culture pool #** |
| --- | --- | --- | --- | --- | --- | --- |
| chr1:16152796 | AATGCTGGCTCGACAGGCTG | AGG | Intergenic | 0 - 0 - 0 - 12 | 4, 7, 9 | 1 |
| chr10:70092203 | AATTCAGTGGACGACGCCGA | GGG | PBLD intronic | 0 - 0 - 0 - 1 | - |  |
| chr12:10876057 | TCATTAGCATTTAAAGGCGC | CGG | Intergenic | 0 - 0 - 0 - 8 | 4, 7, 9 |  |
| chr12:53815822 | TCGACCCCTTCGGCCGGGCG | CGG | Intergenic | 0 - 0 - 1 - 8 | 4, 7, 9 |  |
| chr12:58246976 | TTCTTGAGGCCAGAACGAAG | CGG | Intergenic | 0 - 0 - 4 - 23 | 4, 7, 9 |  |
| chr12:92863772 | CACGTGGACAGGGCTGAAGC | CGG | LINC02397 | 0 - 0 - 4 - 23 | - |  |
| chr12:106150227 | AATTAGCCGGAGTGGTGGTG | GGG | Intergenic | 0 - 0 - 33 - 48 | - |  |
| chr12:128055569 | ACATGGTGCCCCGTCGGCTA | CGG | Intergenic | 0 - 0 - 0 - 2 | 7, 9 |  |
| chr12:130765849 | TGGGCCCAGGCTCGGGGGCT | GGG | Intergenic | 0 - 0 - 7 - 50 | 7, 9 |  |
| chr14:30809300 | AATCATGATGTCTGTCTTCA | TGG | Intergenic | 0 - 0 - 3 - 63 | 7, 9 | 2 |
| chr14:39901580 | CAGCGGCCCGGAAGCCTCAA | GGG | FBXO33 UTR | 0 - 0 - 0 - 5 | 9 |  |
| chr14:42234257 | TTTCAAGACGTTAAAGAAAC | AGG | LRFN5 intronic | 0 - 0 - 5 - 33 | 9 |  |
| chr14:69405738 | GCCAAGAAGCAGGGGGCCTG | CGG | ACTN1 intronic | 0 - 0 - 7 - 39 | - |  |
| chr14:75084005 | TTTGGAAGGTGCAGGCCGTA | CGG | Intergenic | 0 - 0 - 1 - 3 | - |  |
| chr14:75780274 | CCACAAAGTACACAAAGAAC | AGG | Intergenic | 0 - 0 - 5 - 23 | - |  |
| chr14:91885563 | TTAATTGCTTCTCCGCCCGC | CGG | Intergenic | 0 - 0 - 0 - 1 | - |  |
| chr16:14136787 | GGCTTTGTTTATGGGACAGA | TGG | Intergenic | 0 - 0 - 3 - 21 | - |  |
| chr16:55468556 | AACCCCGGCCACCCAGGCTG | GGG | MMP2-AS1 intronic | 0 - 0 - 13 - 106 | - |  |
| chr16:58087434 | ACGTGGTCAGCCACCACAGA | CGG | Intergenic | 0 - 0 - 1 - 14 | - | 3 |
| chr16:75121047 | AGAGTTCTTGTAGCTTGAAC | CGG | ZNRF1 intronic | 0 - 0 - 2 - 14 | - |  |
| chr16:78133950 | GACCCGAGGCCCACCAAGGG | GGG | WWOX UTR | 0 - 0 - 1 - 17 | - |  |
| chr16:79964797 | AGGCCAAGTGGATGGATGAT | GGG | Intergenic | 0 - 0 - 4 - 25 | - |  |
| chr16:89336911 | GTGAGCCCCCTCTAAGGTCC | CGG | ANKRD11 intronic | 0 - 0 - 2 - 5 | - |  |
| chr17:29776374 | TCCTTTCCATTCGCTTCTGG | AGG | RAB11FIP4 intronic | 0 - 0 - 0 - 9 | - |  |
| chr17:38082034 | GCATCTCTTCAATCAGAATG | CGG | ORMDL3 exonic | 0 - 0 - 1 - 22 | - |  |
| chr17:44928421 | AACCGCTCTCTGGAAGCGGG | GGG | Intergenic | 0 - 0 - 1 - 3 | - |  |
| chr17:71383391 | ATTTAAAAAAGAGAGGCCAG | GGG | SDK2 intronic | 0 - 0 - 15 - 75 | - |  |

^*^CRISPOR analysis of the sgRNAs to identify the potential perfect and off-target sites (1-2-3 mismatches) in the hg38 human reference genome.

**Table S9. sgRNAs used in co-cultures (quad, pool) to test selectivity toxicity of Panc10.05-specific sgRNAs.**

| **Experiment** | **Target in Panc10.05** | **sgRNA sequence** | **PAM** | **Location type** | **Potential off-target sites (0-1-2-3 mismatches) in hg38^1^** |
| --- | --- | --- | --- | --- | --- |
| NT quad, pool | Non-targeting (negative control)^2^ | GGAATCATCTTCACAGTTGT | NA | NA | 0-0-4-28 |
|  |  | AATATCCTGCCACCTCTAAC | NA | NA | 0-1-0-8 |
| NT quad |  | CCTCCGTCAGCAGCTAACCC | NA | NA | 0-0-0-9 |
|  |  | ACAGATGGAGCCCAACAGAG | NA | NA | 0-0-1-35 |
| NT pool |  | TCAGTCCAGTCAAAGGTGGA | NA | NA | 0-0-3-15 |
|  |  | CTAATGTATGACTGAAAGCT | NA | NA | 0-0-1-19 |
| Panc10.05-specific quad, pool | chr13:67159869 | GAGTGGCCTGTGATGACACT | GGG | PCDH9 intronic | 0-0-1-19 |
|  | chr13:98046823 | GCAGAAAGAGATAGAATGGT | GGG | Intergenic | 0-0-3-52 |
|  | chr3:67534507 | GGGCCTTACCTGAAAGCAGC | AGG | SUCLG2 intronic | 0-0-1-23 |
|  | chr3:76973237 | AGAATTTGAGCGACAGTATG | TGG | ROBO2 intronic | 0-0-2-9 |
| Pool | 12-target | TTGTCCCACAATGATACTTG | NGG | Intergenic regions | 12-8-1-8 |
| Pool | Repetitive regions (positive control) | AGGAGGAGGAGGAGGAGGAG |  | Trinucleotide repeats | >100 perfect target sites |
|  |  | TGCCTCACCTGGGAAGCGCA |  | LINE-1 |  |
| Quad, pool |  | TTGCCCAGGCTGGAGTGCAG |  | Alu elements |  |

1. CRISPOR analysis of the sgRNAs to identify the potential perfect and off-target sites (1-2-3 mismatches) in the hg38 human reference genome. 2. NA: not applicable.

**Table S10. sgRNAs used in co-cultures to test selectivity toxicity of Panc480-specific sgRNAs.**

| **Pool #** | **Target** | **sgRNA sequence** | **PAM** | **Location type** | **Potential off-target sites (0-1-2-3 mismatches) in hg38^*^** |
| --- | --- | --- | --- | --- | --- |
| NT pool | NA | GGACCTGGACTTCTTCAAGG | NA | NA | 0-0-0-17 |
|  | NA | GAAGTGAGATTCAACCATAA | NA | NA | 0-0-0-10 |
|  | NA | CGGGTAAGAGGGAAGGATAA | NA | NA | 0-0-1-24 |
|  | NA | GAGTGGCCTGTGATGACACT | NA | NA | 0-0-1-15 |
| 1 | chr8:201457 | GGAATCATCTTCACAGTTGT | TGG | ZNF596 intronic | 0-0-4-28 |
|  | chr17:5377742 | AATATCCTGCCACCTCTAAC | AGG | DERL2 intronic | 0-1-0-8 |
|  | chr3:537601 | TCAGTCCAGTCAAAGGTGGA | AGG | Intergenic | 0-0-3-15 |
|  | chr3:59525282 | CTAATGTATGACTGAAAGCT | GGG | Intergenic | 0-0-1-19 |
| 2 | chr3:59525282 | CTAATGTATGACTGAAAGCT | GGG | Intergenic | 0-0-1-19 |
|  | chr11:64364029 | TTGGTCATTGGGACCACTGT | AGG | Intergenic | 0-0-0-10 |
|  | chr18:1819017 | TTAGGGGGCCAAGAGCGTAT | GGG | LINC00470, METTL4 intronic | 0-0-0-3 |
|  | chr19:58564841 | GAAGCCGAGTATCTGGGACG | AGG | ZSCAN1 intronic | 0-0-0-2 |
| 3 | chrX:9499045 | CCTCCGTCAGCAGCTAACCC | TGG | Intergenic | 0-0-0-9 |
|  | chrX:23403852 | ACAGATGGAGCCCAACAGAG | GGG | Intergenic | 0-0-1-35 |
|  | chr19:5683012 | CGTGCTGGTGCACCCACTTG | TGG | Intergenic | 0-0-0-9 |
|  | chr19:18760457 | GCCGGCAGCGGGAACCCAGC | CGG | KLHL26 5’UTR | 0-0-2-29 |
| Positive control pool | 12-target | TTGTCCCACAATGATACTTG | NGG | Intergenic regions | 12-8-1-8 |
|  | Repetitive regions | AGGAGGAGGAGGAGGAGGAG |  | Trinucleotide repeats | >100 perfect target sites |
|  |  | TGCCTCACCTGGGAAGCGCA |  | LINE-1 |  |
|  |  | TTGCCCAGGCTGGAGTGCAG |  | Alu elements |  |

^*^CRISPOR analysis of the sgRNAs to identify the potential perfect and off-target sites (1-2-3 mismatches) in the hg38 human reference genome.

**Table S11. Cutting efficiency and number of potential off-target sites of sgRNAs included in Panc480-MT7.**

| **Target** | **sgRNA sequence** | **PAM** | **Mutation type (copy number)** | **Mutation frequency (%)^&^** | **No. of potential off-targets (0-1-2-3-4mm)^*^** | **No. of potential off-targets including NAG PAM^$^** |
| --- | --- | --- | --- | --- | --- | --- |
| chr8:201457 | GGAATCATCTTCACAGTTGT | TGG | D-LOH^#^ (1) | 22.6 | 0-0-3-20-159 | 1-0-3-44-376 |
| chr17:5377742 | AATATCCTGCCACCTCTAAC | AGG | D-LOH (1) | 36.4 | 0-0-0-6-96 | 1-1-0-16-234 |
| chr3:537601 | TCAGTCCAGTCAAAGGTGGA | AGG | D-LOH (1) | 87.3 | 0-0-1-7-119 | 0-0-3-26-281 |
| chr3:59525282 | CTAATGTATGACTGAAAGCT | GGG | D-LOH (1) | 71.1 | 0-0-1-7-137 | 0-0-1-22-359 |
| chrX:3982448 | GAGGTGTCTAAACCATGACA | AGG | D-LOH (1) | 67.8 | 0-0-0-4-103 | 1-0-1-15-230 |
| chr8:29032916 | GTGCACATCTTATCTCCCTT | AGG | D-LOH (1) | 57.6 | 0-0-0-8-117 | 0-1-1-14-271 |
| chr18:1819017 | TTAGGGGGCCAAGAGCGTAT | GGG | D-LOH (1) | 68.7 | 0-0-0-2-32 | 0-0-0-5-67 |

^#^D-LOH: deletion-based loss of heterozygosity

^&^Individual sgRNAs were transduced into Panc480 cells separately and puromycin-selected for 7 days. Cells were harvested for NGS and mutation frequency was quantified using CRISPResso2.

^*^sgRNA sequences were put through Cas-OFFinder to identify potential off-target sites with 1-4 mismatches in hg19. Only sites with the canonical NGG PAM were included.

^$^sgRNA sequences were put through Cas-OFFinder to identify potential off-target sites including ones with non-canonical NAG PAM and ones with 1-4 mismatches.
